# Perovskite ferroelectric tuned by thermal strain

**DOI:** 10.1038/s41598-019-40260-y

**Published:** 2019-03-06

**Authors:** M. Tyunina, O. Pacherova, J. Peräntie, M. Savinov, M. Jelinek, H. Jantunen, A. Dejneka

**Affiliations:** 1 Microelectronics Research Unit, University of Oulu, P.O. Box 4500, FI-90014 Oulu, Finland; 2 Institute of Physics of the Czech Academy of Sciences, Na Slovance 2, 18221 Prague, Czech Republic

## Abstract

Modern environmental and sustainability issues as well as the growing demand for applications in the life sciences and medicine put special requirements to the chemical composition of many functional materials. To achieve desired performance within these requirements, innovative approaches are needed. In this work, we experimentally demonstrate that thermal strain can effectively tune the crystal structure and versatile properties of relatively thick films of environmentally friendly, biocompatible, and low-cost perovskite ferroelectric barium titanate. The strain arises during post-deposition cooling due to a mismatch between the thermal expansion coefficients of the films and the substrate materials. The strain-induced in-plane polarization enables excellent performance of bottom-to-top barium titanate capacitors akin to that of exemplary lead-containing relaxor ferroelectrics. Our work shows that controlling thermal strain can help tailor response functions in a straightforward manner.

## Introduction

Strong dielectric, piezoelectric, pyroelectric, electrooptic, electrocaloric, and other response functions of perovskite *AB*O_3_-type oxide ferroelectrics (FEs) enable numerous commercialized and emerging advanced applications^1–3^. The key fundamental property of FEs is large polarization, which is switchable by an electric field. However, the largest mainstream application is in capacitors, employing the very high dielectric permittivity of FEs. Billions of multilayer FE ceramic capacitors are produced annually. Apart from ceramics technologies, progress in synthesis of FE thin films allows for integrated and low-voltage FE applications^4,5^. FE thin-film capacitors are utilized to achieve extremely high capacitance densities, in tunable radio-frequency circuits, and in random-access memory devices. Dedicated capacitors make use of selected best-suited properties of FE thin films. For instance, a high dielectric permittivity or a large and switchable polarization are desirable for memories, while hysteresis-free electric-field-dependent permittivity and low losses are desirable for radio-frequency tunable capacitors. A large maximum polarization, slim polarization-field loops, and an increased breakdown strength are required for storage capacitors^6–10^.

Because cationic composition (*A* and *B* cations) determines properties of perovskite *AB*O_3_ oxides, an adequate route to tailor FE films for specified applications is by varying the cations. Namely, Pb(Zr,Ti)O_3_ (PZT) and BiFeO_3_ (BFO) ensure large polarization; paraelectric (Ba,Sr)TiO_3_ (BST) enables hysteresis-free tunable permittivity; and relaxor FE PbMg_1/3_Nb_2/3_O_3_ (PMN), PbSc_0.5_Nb_0.5_O3 (PSN), or (Pb,La)(Zr,Ti)O_3_ (PLZT) provide high permittivity and slim polarization-field loops. Importantly, modern environmental and sustainability issues motivate the reduction of elements such as Pb, Bi, Sc, and Nb in technologies and products^11^. Beyond these issues, noteworthy FE applications in the life sciences and medicine require biocompatible FEs that place additional restrictions on cationic composition^12,13^. Therefore, novel routes to engineer FE properties are highly relevant today. In particular, heteroepitaxial growth makes it possible to create lattice strain, leading to significant changes in the phase diagrams and functions of FEs^14^. Epitaxial in-plane strain arises from the misfit between lattice parameters on a substrate surface and those of a film material. We note that a relatively narrow selection of suitable single-crystal substrates and a small critical thickness, at which the relaxation of misfit strain starts (a few to tens of nanometers), mitigate the wide utilization of misfit-strained films.

Here, we show that thermal strain can efficiently tune the performance of FE thin films for versatile capacitors. Because the deposition of perovskite-structure oxides requires temperatures of 800–1200 K, the mismatch between the coefficients of thermal expansion (CTE) of the film and the substrate materials causes a build-up of film lattice strain during post-deposition cooling. The linear CTE values are positive and typically (0.6–1.5) × 10^−5^ K^−1^ in the high-temperature cubic paraelectric phase of many FEs and ∼(0.7–1.3) × 10^−5^ K^−1^ in common oxide substrates^15–20^. These coefficients suggest possible in-plane compressive or tensile thermal-mismatch strain (or thermal strain here for brevity) up to 1% in magnitude for FE films on oxide substrates. In contrast to misfit strain, thermal strain can be varied continuously by altering deposition temperature. Additional degrees of freedom in controlling thermal strain come from Si substrates (CTE 0.26 × 10^−5^ K^−1^ at 300 K and 4.2 × 10^−5^ K^−1^ at 1000 K) and buffer layers^21^.

We demonstrate thermal strain leading to novel properties in relatively thick (100–200 nm) films of archetypical, environmentally friendly, biocompatible, and low-cost FE BaTiO_3_ (BTO)^22^ on common oxide substrates. The film-substrate compressive misfit strain is large and relaxes during high-temperature deposition. During cooling, the tensile thermal strain builds up. The thermal tension produces in-plane polarization, which rotates under the application of an out-of-plane electric field. This polarization behavior leads to BTO thin-film capacitors, which are competitive with analogous capacitor stacks using high-performance but environmentally unfriendly and costly relaxor PSN.

The thermally strained 200-nm-thick BTO films ensure an excellent capacitance density of ∼3 μF/cm^2^ changing by less than 13% only in the temperature range of 250–400 K, a high dielectric tunability up to 65% at 4 V, slim polarization-field loops with a maximum polarization of 18 μC/cm^2^ at 12 V, and a stored energy density up to 5 J/cm^3^ with a storage efficiency up to 65% at 12 V.

## Results and Discussion

### Thermal strain

**Figure 1:**
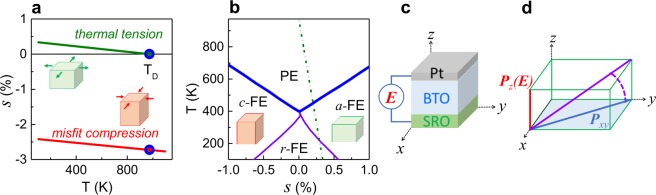
Theoretical (**a**) compressive misfit strain and tensile thermal strain as a function of temperature in BTO on STO and (**b**) strain-temperature phase diagram in a BTO film. The dashed line shows the thermodynamic path for BTO on STO. BTO unit cells are shown schematically. Schematics of (**c**) the SRO/BTO/Pt bottom-to-top capacitor under an applied electric field and (**d**) the electric-field induced rotation of the BTO polarization in such a capacitor.

Figure 1 illustrates our approach. The BTO misfit strain is [*s*_*a*_ = *a*_*S*_/*a*_*0*_−1], where *a*_*S*_ is the lattice parameter on the surface of underlying substrate or bottom layer and *a*_*0*_ is the lattice parameter of a cubic cell of BTO (*a*_*0*_ = *V*^1/3^ for unit-cell volume *V*). The theoretical misfit strain is estimated in the temperature range of 100–1100 K using the published and linearly extrapolated lattice constants of STO, DSO, and BTO^18,20^. The misfit strain is compressive, with its magnitude larger than 2.5% on STO (Fig. 1a) and 1.5% on DSO (Supplementary Fig. S1). The corresponding critical thickness is only a few nanometers^23,24^, suggesting a relaxation of misfit strain during the deposition of the BTO films of 100–200 nm in thickness.

Assuming complete misfit relaxation during film growth at the deposition temperature *T*_*D*_ = 973 K and using the CTEs of the substrate (*α*_*S*_) and BTO (*α*_*B*_), and the substrate and BTO lattice parameters *a*_*S*_ = *a*_*0S*_ (1 + *α*_*S*_*T*) and *a*_*0*_ = *a*_*00*_ (1 + *α*_*B*_*T*), the thermal strain *s*_*T*_ in the BTO films is estimated as

1 $${s}_{T}\approx \frac{({\alpha }_{B}-{\alpha }_{S}\frac{{a}_{{0S}}}{{a}_{{00}}})}{(1+{\alpha }_{B}T)}(T-{T}_{D}).$$

The parameters *a*_*0S*_ and *a*_*00*_ (3.894 Å for STO, 3.931 Å for DSO, and 3.989 Å for BTO) and the coefficients *α*_S_ and *α*_B_ (0.88×10^−5^ Å^−1^ for STO, 1.12×10^−5^ Å^−1^ for DSO, and 1.25×10^−5^ Å^−1^ for BTO) are extracted from refs^18,20^. The expected thermal strain is in-plane tensile, opposing misfit compression, in BTO on STO (Fig. 1a) and DSO (Supplementary Fig. S1).

The calculated temperature-dependent thermal strain is then considered within the theoretical strain-temperature phase diagram of BTO (schematics in Fig. 1b)^25–27^. The BTO tensile thermal strain increases upon cooling, as illustrated by the thermodynamic path (dashed line in Fig. 1b). The path crosses the phase boundary (thick line), meaning that the film experiences a phase transition from a high-temperature paraelectric (PE) to an FE *a*-phase, possessing nonzero in-plane and zero out-of-plane components of polarization^25–27^. This *a*-phase contrasts with an FE *c*-phase, where compressive strain produces a nonzero out-of-plane polarization only. Upon further cooling, an out-of-plane polarization component additionally appears at the transition from the *a*- to an *r*-FE phase (the thermodynamic path crosses the *a*FE-*r*FE boundary in Fig. 1b). We note that abrupt changes of the lattice parameters at the PE-*a*FE and *a*FE-*r*FE transitions should be absent^25–27^.

The thermal tension enforces the polarization to lie in the plane parallel to the substrate surface in the BTO films (*P*_xy_ in Fig. 1d). When such films are sandwiched between bottom and top electrodes (Fig. 1c), the electric field (*E*) is applied along the out-of-plane direction, which is nonpolar in the films. The out-of-plane electric field is expected to induce out-of-plane component of polarization (*P*_z_ in Fig. 1d), resulting in rotation of the total polarization (shown schematically by the dashed curve in Fig. 1d).

Here, we prepare thermally strained BTO films and demonstrate a very high and thermally stable capacitance density and good storage characteristics, associated with such polarization rotation therein.

### Films

Thin films of BTO and reference PbSc_0.5_Nb_0.5_O3 (PSN) were deposited using SrRuO_3_ (SRO) bottom electrode layer or without SRO on (001) SrTiO_3_ (STO) and (011) DyScO_3_ (DSO) substrates. The films were grown by pulsed laser deposition (PLD) at a substrate temperature of 973 K and a pressure of ambient oxygen of 20 Pa. The employed high oxygen pressure ensures the growth of stoichiometric defect-free BTO films, in contrast to defect formation at lower pressures^28,29^.

**Figure 2:**
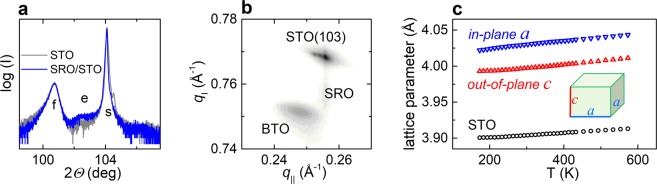
XRD analysis. (**a**) Details of the *Θ*-2*Θ* scans around the (004) perovskite diffraction peaks in the 100-nm-thick BTO films on STO and SRO-coated STO. Diffractions from the BTO films, substrates, and SRO are marked by “f”, “s”, and “e”, correspondingly. (**b**) Reciprocal space map around (103) perovskite diffraction in the BTO/SRO/STO stack. (**c**) Lattice parameters as a function of temperature in the BTO film on STO and in the STO substrate.

To verify the presence and evolution of thermal strain in our BTO films, the crystal structure and lattice parameters of the films were studied by XRD. The room-temperature *Θ*–2*Θ* scans and reciprocal space maps (Fig. 2b and Supplementary Fig. S2) reveal that the BTO films are highly-oriented perovskite with (00 *l*) planes parallel to the (001)STO or (011)DSO substrate surfaces. Thin bottom SRO electrode layers grow pseudomorphically on STO and DSO, which is consistent with the previous observations^30^. Cube-on-cube-type epitaxial relationships in terms of perovskite cells are [100](001)BTO || [100](001)STO, [100](001)BTO || [100](001)SRO || [100] (001)STO, and [100](001)BTO || [100](001)SRO || [110](011)DSO. The in-plane lattice parameters *a* of the BTO films are equal to each other and exceed the out-of-plane parameter *c*: *a* > *c*. The unit cells of BTO can be described as metrically tetragonal with a negative tetragonality (*c*/*a* − 1) < 0. For the 100-nm-thick BTO films on bare STO and on STO coated with SRO 20 nm thick, the lattice parameters are approximately the same (Fig. 2a), implying a minor influence of the very thin SRO. The BTO in-plane parameters are 4.031 Å on SRO/STO and 4.022 Å on SRO/DSO. The BTO in-plane strain, evaluated as *s* = (*a*/*a*_0_ − 1), is tensile ∼0.6% on STO and ∼0.4% on DSO. The out-of-plane parameters are 3.993 Å on STO and 3.990 Å on DSO. Thus, the misfit compressive strain is relaxed and the thermal tensile strain is present in the BTO films. Compared to bulk BTO, the unit cells of the films are expanded in precise agreement with the in-plane biaxial tension and elastic constants of BTO. Because anomalous lattice expansion is typical for defect-rich films (Supplementary Fig. S3)^29^, these results indicate proper stoichiometry of our thermally strained BTO films.

Interestingly, with increasing the film thickness to 200 nm (BTO) and 60 nm (SRO), the BTO tension changes to 0.3% (*a* = 4.018 Å and *c* = 3.998 Å) (Supplementary Fig. S2c). The SRO expansion should not significantly affect the thermal strain in BTO on SRO/STO as the CTEs of SRO and STO are approximately the same^30^. However, the total stress is distributed between SRO and BTO due to equal traction on both sides at the SRO-BTO interface^31,32^. Because of the BTO-SRO elastic coupling, the strain state of BTO can be fine-tuned by varying the film thicknesses. More generally, thermal strain can be widely altered using different materials and thicknesses of the bottom buffer and/or electrode layers.

To further validate the thermal tension, the lattice parameters of the BTO film (100 nm) on STO were measured as a function of temperature (Fig. 2c). The film parameters smoothly decrease on cooling, in contrast to the abrupt changes at the phase transitions in bulk BTO. A minor deviation from the linear temperature dependence below 200 K is caused by the changes in the STO substrate (Supplementary Fig. S4). The film behavior agrees with the theoretical PE-*a*FE and *a*FE-*r*FE transitions induced by tensile strain, as explained above (see the strain temperature *s*-*T* phase diagram in Fig. 1b). Thus, the thermal strain induces peculiar monoclinic-type crystal phases, in resemblance with action of misfit strain.

### Capacitors

**Figure 3:**
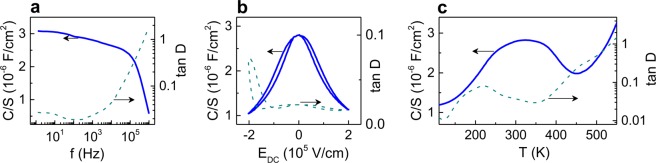
Capacitance density and loss factor as a function of (**a**) frequency, (**b**) dc electric field, and (**b**) temperature. Measurements were performed at temperature of 300 K (**a**,**b**) and frequency 1 kHz (**b**,**c**).

The obtained typical room-temperature capacitance density C/S is ∼3 μF/cm^2^ (corresponding to the dielectric permittivity of ∼700) with losses tan *D* < 0.02 (Fig. 3a). It is known that an impedance of parallel-plate ferroelectric thin-film capacitors includes contributions from the dielectric permittivity and dielectric losses of the ferroelectric films, from the resistance of electrodes, and from the resistance of the film^33^. Therefore, the permittivity and loss measured in the capacitor are not the same as those of the ferroelectric film itself. Often, the measured permittivity (loss) in capacitors is smaller (larger) compared to that in the film itself mainly due to the presence of thin-film electrodes^33^. Because of this presence, also a drop in capacitance and an increase in losses occur at high frequencies^33^, which are above 100 kHz here. For comparison, a capacitor of an analogous type with a 200-nm-thick epitaxial PSN film exhibits a larger permittivity but lower cut-off frequency and significantly higher losses (Supplementary Figs S5 and S6).

The BTO capacitor provides excellent tunability under an applied DC electric field (*E*_*DC*_) (Fig. 3b). The tunability of capacitance is defined as {*δC* = [1 − *C*(*E*_*DC*_)/*C*(0)]}, where *C*(0) and *C*(*E*_*DC*_) are the capacitances at zero and nonzero field *E*_*DC*_, correspondingly. For *E*_*DC*_ = 2 × 10^5^ V/cm (at a bias voltage of 4 V only), the BTO tunability is 63%. The figure of merit [FOM = *δC*/(tan *D*)] is ∼31–32 in the BTO stack compared to ∼8 only in the PSN capacitor (Supplementary Fig. S7). Also for comparison, the tunability and FOM are ∼40–50% and ∼6–16, correspondingly, in the (Ba,Sr)TiO_3_/SRO capacitors^34^. Because of the in-plane polarization, the dielectric hysteresis is minor in the BTO capacitor and similar to that in the relaxor PSN capacitor (Supplementary Fig. S7).

Importantly, the BTO capacitors possess remarkable thermal stability owing to the thermal-strain-induced *a*FE and *r*FE phases. The *a*FE-*r*FE phase transition produces a broad peak in capacitance (permittivity) around *T*_*m*_ ≈ 323 K (Fig. 3c). The PE-*a*FE transition occurs at *T* > 450–500 K. The two corresponding peaks in losses also manifest these transitions. The dielectric anomalies in BTO are consistent with the PE-*a*FE and *a*FE-*r*FE phase transitions in the *s*-*T* diagram (Fig. 1b). The resulting thermal variation of capacitance Δ*C*_T_ = [1 − *C*_*min*_/*C*_*max*_] is ≤ 13% only in the temperature range of 250–400 K. For comparison, the capacitance variation is ∼25% for the same temperatures in the PSN capacitor (*T*_*m*_ = 335 K, Supplementary Fig. S8).

**Figure 4:**
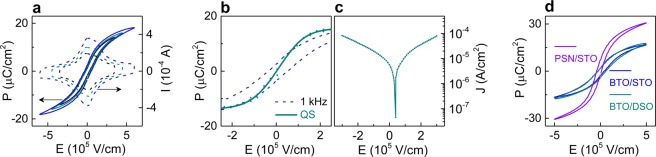
Dynamic (**a**) polarization-field and current-field hysteresis loops, (**b**) quasi-static (marked by QS) and dynamic (dashed line) polarization-field loops, and (**c**) leakage current in SRO/BTO/Pt. (**d**) Dynamic hysteresis loops in the BTO and PSN capacitors. Dynamic loops were measured at frequency 1 kHz.

Thermal tension induces the in-plane polarization in the *a*FE phase of BTO. An out-of-plane component of polarization appears under the action of an electric field applied to the SRO/BTO/Pt capacitor (Fig. 1d). Due to such polarization rotation, the BTO capacitors demonstrate slim dynamic polarization-field (*P*-*E*) hysteresis loops (Fig. 4a,b), contrasting with conventional FE hysteresis and resembling relaxor behavior. The lack of conventional FE hysteresis is confirmed by the current-field (*I*-*E*) loops exhibiting peaks only near the zero field. We stress that the *P*-*E* curves measured under quasi-static conditions are completely hysteresis-free (Fig. 4b). Both the quasi-static and dynamic measurements show the field-induced out-of-plane polarization. The quasi-static loops have zero coercive field. The dynamic loops exhibit non-zero coercive fields, which depend on maximum applied field. In particular, the presence of mobile charge carriers may lead to a broadening of the dynamic loops (Supplementary Fig. S9)^35,36^. Here, the density *J* of the leakage current increases with the field (Fig. 4c). Closer inspection reveals a combination of a Schottky-type injection of carriers at fields up to 10^5^ V/cm (ln*J* ∝ √*E*) and space charge limited conduction for stronger fields (*J* ∝ *E*^2^) (Supplementary Fig. S10)^37^. Interestingly, a lower injection barrier is responsible for a larger current density at negative bias compared to a positive bias, which is consistent with the asymmetry in losses (Fig. 3b). Thus, the losses and dynamic hysteresis may be further reduced by increasing injection barriers, mainly through improvements in electrodes and/or film-electrode interfaces.

The *P-E* loops are similar in the BTO capacitors on the STO and DSO substrates (Fig. 4d) despite slightly different in-plane BTO strains therein. This observation corroborates the electric-field-induced nature of the out-of-plane polarization component, which is responsible for the loops. We stress that, although the BTO loops resemble those in PSN (Fig. 4d), the mechanisms behind them differ: the polarization rotation in BTO opposes field-activated flips of random polar entities (giant dipoles) in relaxor PSN^38,39^.

Relaxor materials such as PSN are of particular interest for advanced energy storage applications because of slim *P-E* loops and a rather large polarization. Owing to slim loops, whose remnant polarization *P*_*r*_ and coercive field are very weak functions of the maximum applied voltage (Fig. 4a), the thermally strained BTO films are competitive with the PSN films for such applications in the low-voltage range.

As accepted, the stored energy density *U*_*store*_, recoverable energy density *U*_*recov*_, and storage efficiency *η* were determined from the dynamic *P-V* loops acquired at 1 kHz as a function of maximum applied voltage:

2 $${U}_{store}={\int }_{{P}_{r}^{-}}^{{P}_{max}}EdP,$$

3 $${U}_{recov}={\int }_{{P}_{r}^{+}}^{{P}_{max}}EdP,$$

4 $$\eta =\frac{{U}_{recov}}{{U}_{store}}\cdot 100\, \% {\rm{.}}$$

**Figure 5:**
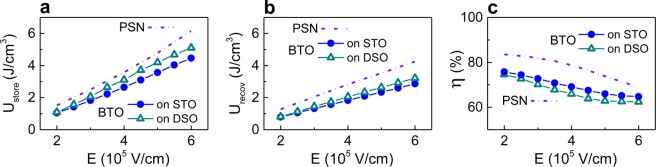
(**a**) Stored energy density, (**b**) recoverable energy density, and (**c**) storage efficiency in the BTO and PSN capacitors. Finally, we note that also polycrystalline FE films often exhibit relatively slim and sloped polarization-field loops. However, compared to the thermally strained BTO films, significantly smaller polarization and larger coercive fields are obtained in polycrystalline BTO films (Supplementary Fig. S13).

The dynamic storage characteristics of the BTO and PSN capacitors are very similar at *V* ≤ 12 V (Fig. 5). To compare these characteristics with the data on other thin-film capacitors, we linearly extrapolate the results in Fig. 5 to a field magnitude of 10^6^ V/cm. The extrapolated efficiency η ≈ 65% and recoverable density *U*_*recov*_ ≈ 5 J/cm^3^ of the BTO capacitor are within the ranges reported for many high-performance Pb-containing and Pb-free thin films^40–42^. We note that quasi-static density *U*_*recov*_ is larger than that at 1 kHz and that quasi-static *U*_*recov*_ = *U*_*store*_ leading to η = 100% (Supplementary Fig. S11). Moreover, our BTO capacitors exhibit very high capacitance density and tunability. Additionally, the BTO capacitors demonstrate good fatigue behavior: the maximum polarization and dielectric permittivity remain unchanged for 10^6^ switching cycles and decrease by less than 5% after 10^9^ cycles (Supplementary Fig. S12).

Our work shows that BTO properties can be substantially tuned by thermal strain in relatively thick films. Because lattice strain affects properties in a broad range of materials^43^, controlling thermal strain enriches the palette of tools to create dedicated functionalities at will. Importantly, thermal strain can be continuously varied by altering synthesis temperature. Additionally, thermal strain can be adjusted by using pertinent buffer layers of various materials and thicknesses. Therefore, precise engineering of thermal strain is feasible and ensures unique control of materials performance.

## Conclusions

We have experimentally demonstrated that the versatile properties of relatively thick (200 nm) films of environmentally friendly, biocompatible, and low-cost ferroelectric BaTiO_3_ can be tailored by thermal strain arising from a mismatch between the thermal expansion coefficients of the substrates and BaTiO_3_. The film thermal tension builds up during cooling from a high synthesis temperature and results in the in-plane orientation of ferroelectric polarization. Owing to the in-plane polarization and the electric-field-induced polarization rotation, bottom-to-top BaTiO_3_ capacitors exhibit excellent performance and are competitive with those of the exemplary but environmentally unfriendly and expensive relaxor PbSc_0.5_Nb_0.5_O_3_. We anticipate that thermal strain can effectively tune the performance of many materials.

## Methods

Thin films of BTO and reference PSN were deposited using SrRuO_3_ (SRO) bottom electrode layer or without SRO. Single-crystal epitaxially polished (001) SrTiO_3_ (STO) and (011) DyScO_3_ (DSO) substrates were purchased from MTI Corporation. The films were grown by pulsed laser deposition (PLD) using a KrF excimer laser (energy density ∼2 J/cm^2^) at a substrate temperature of 973 K and a pressure of ambient oxygen of 20 Pa. The employed high oxygen pressure ensures the growth of stoichiometric defect-free BTO films, in contrast to defect formation at lower pressures^28,29^. To further secure a proper oxygen stoichiometry, the oxygen pressure was gradually increased to 800–1000 Pa during postdeposition cooling, conducted at a rate of 5 K/min. Capacitor stacks were formed using bottom SRO electrode layer and circular top Pt contact pads (area 0.2 mm^2^), created by room-temperature vacuum PLD of Pt through a shadow mask.

The crystal structure of the films was studied by high-resolution X-ray diffraction (XRD) on D8 DISCOVER diffractometers (Bruker corporation) using Cu Kα radiation. The measurements in the temperature range of (173–573) K were carried out at different fixed temperatures using an ANTON PAAR equipment: DSC 350 cooling stage, TCU 100 temperature controller, and LNC nitrogen suction. The Θ–2Θ scans in the range of 2Θ = (10–130) deg and reciprocal space maps (RSM) in the vicinity of the perovskite (002), (303), and (103) diffractions were acquired. The in-plane (parallel to substrate surface) and out-of-plane (normal to substrate surface) lattice parameters were estimated from the positions of diffraction maxima using substrates as a reference.

The impedance of the capacitors was measured by a NOVOCONTROL Alpha-AN High Performance Frequency Analyzer. The frequency and amplitude of the sinusoidal AC voltage were *f* = (1–10^6^) Hz and *V*_*AC*_ = 1–10 mV, correspondingly. The dielectric hysteresis was inspected by sweeping a biasing DC voltage *V*_*DC*_ superimposed with the probing AC voltage. The measurements were performed on cooling, heating, and at several fixed temperatures. The control of temperature was realized using a JANIS ST-100 He flow cryostat equipped with a LakeShore 335 temperature controller. The temperature was swept at a rate of 3–5 K/min. The polarization and leakage current were measured by a TF 2000E Analyzer (aixACCT Systems GmbH). In all measurements, the electric fields were applied and the response was measured along the out-of-plane direction of the SRO/FE/Pt capacitors. The impedance was analyzed using an equivalent-circuit model of a leaky parallel-plate capacitor connected in series with a thin-film oxide electrode resistor (see, e.g., ref. ^33^. and references therein).

The datasets generated during and/or analysed during the current study are available from the corresponding author on reasonable request.

## Supplementary information

Supporting Information to Perovskite ferroelectric tuned by thermal strain
