# Supplementary material for: Perovskite ferroelectric tuned by thermal strain
Source: Sci Rep. 2019 Mar 6;9:3677. doi: 10.1038/s41598-019-40260-y (PMC6403324; doi:10.1038/s41598-019-40260-y)
Supplement: Supplementary file 1 — Supporting Information to Perovskite ferroelectric tuned by thermal strain [file 41598_2019_40260_MOESM1_ESM.pdf]

Supporting Information

to

Perovskite ferroelectric tuned by thermal strain

M. Tyunina[1,2,*] O. Pacherova[2], J. Peräntie[1], M. Savinov[2], M. Jelinek[2], H. Jantunen[1], A. Dejneka[2]

[1]Microelectronics Research Unit, University of Oulu, P.O. Box 4500, FI-90014 Oulu, Finland

[2]Institute of Physics of the Czech Academy of Sciences, Na Slovance 2, 18221 Prague, Czech Republic

* E-mail: marina.tjunina@oulu.fi

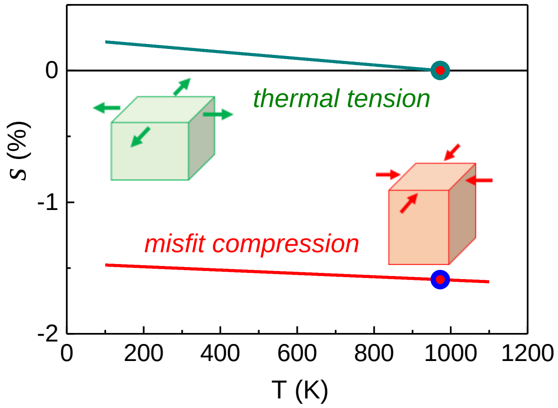

Figure S1. Theoretical compressive misfit strain and tensile thermal strain in BTO on DSO.

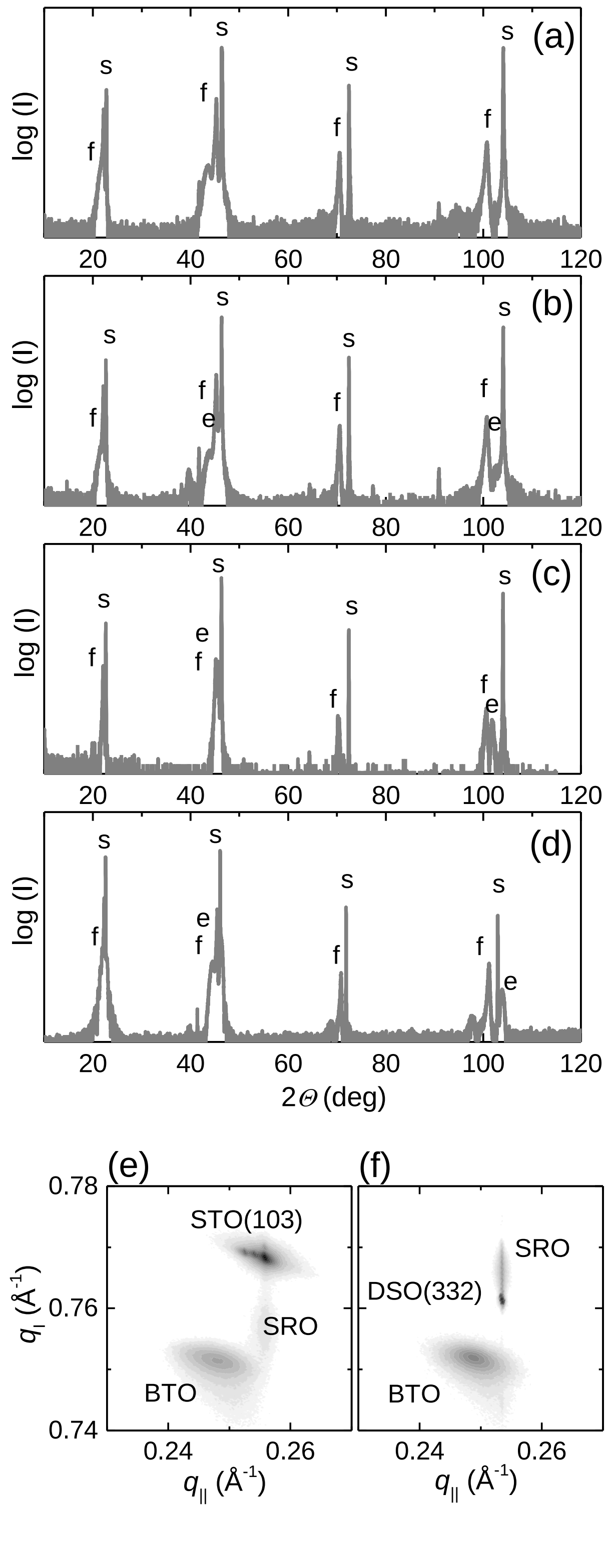

Figure S2. XRD Θ-2Θ scans in (a) BTO(100nm) on STO, (b) BTO(100nm)/SRO(20nm) on STO, (c) BTO(200nm)/SRO(60nm) on STO, (d) BTO(100nm)/SRO(20nm) on DSO. Reciprocal space maps in BTO/SRO on (e) STO and (f) DSO. Diffractions from the BTO films, substrates, and SRO are marked by “f”, “s”, and “e”, correspondingly.

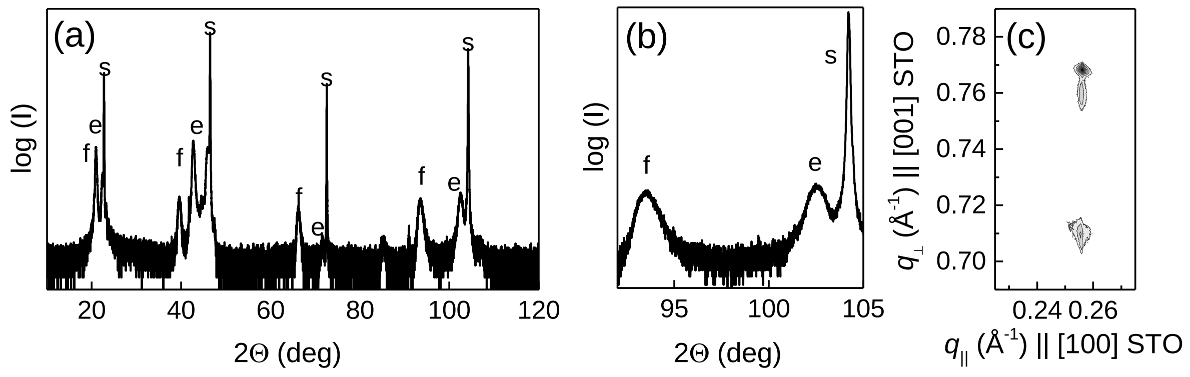

Figure S3. XRD (a, b) Θ–2Θ scans and (c) reciprocal space map in the BTO film deposited at reduced oxygen pressure of 5 Pa using SRO/STO. Diffractions from the BTO films, substrates, and SRO are marked by "f", "s", and "e", correspondingly.

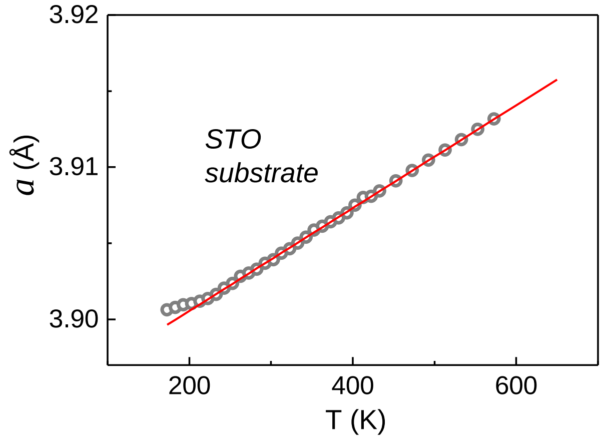

Figure S4. Lattice parameter of the STO substrate as a function of temperature.

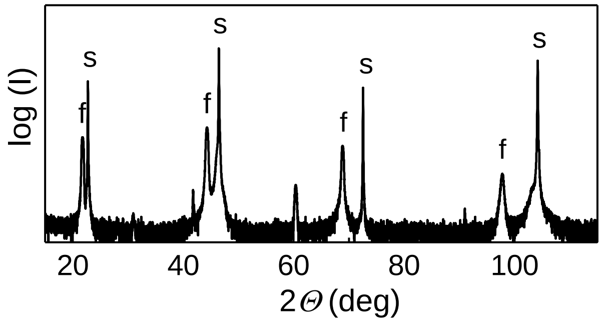

Figure S5. XRD scan in the SRO/PSN(200nm)/Pt stack. Diffraction peaks from the STO substrate and PSN film are marked by "s" and "f", correspondingly.

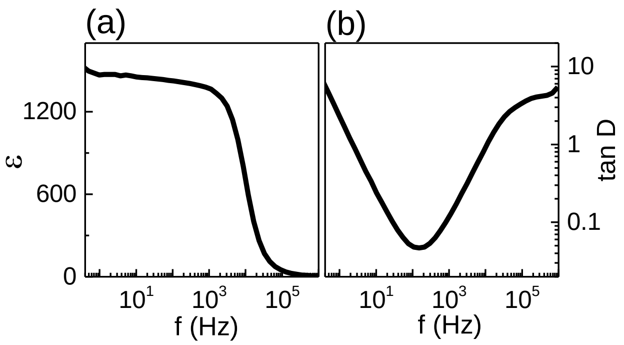

Figure S6. Frequency dispersion of the real part of the dielectric permittivity ε and loss factor tan D at room temperature in the SRO/PSN(200nm)/Pt capacitor.

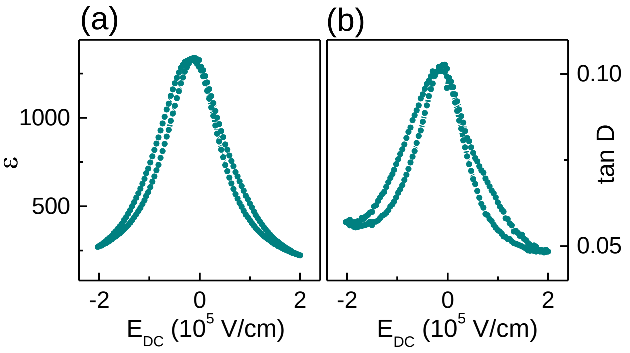

Figure S7. Dielectric permittivity ε and loss factor tan D as a function of dc electric field E_{DC} at room temperature in the SRO/PSN(200nm)/Pt capacitor. Measurement frequency is 1 kHz.

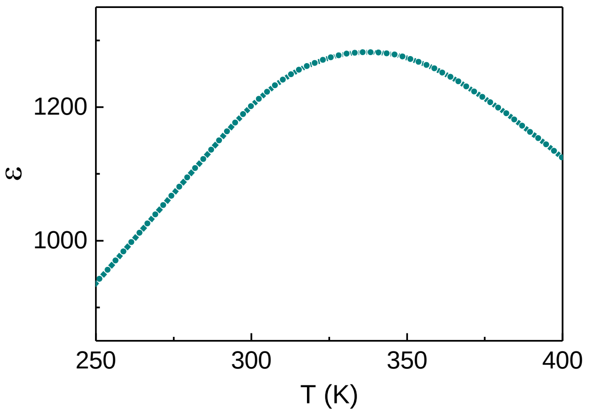

Figure S8. Dielectric permittivity as a function of temperature measured at 1.2 kHz on cooling in the PSN capacitor.

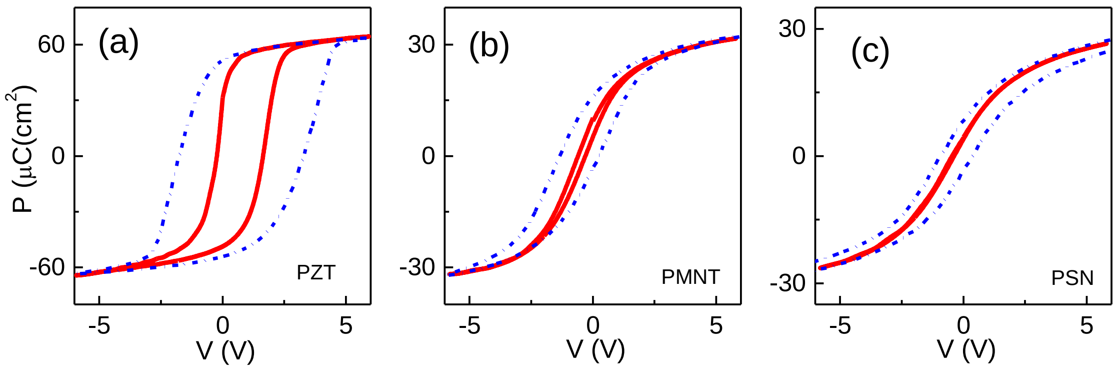

Figure S9. Quasi-static (red solid curves) and dynamic (dashed curves, at 1 kHz) polarization-voltage loops in the 200-nm-thick epitaxial films of (a) $PbZr_{0.2}Ti_{0.8}O_3$, (b) 0.68($PbMg_{1/3}Nb_{2/3}O_3$)-0.32$PbTiO_3$, and (c) $PbSc_{0.5}Nb_{0.5}O_3$ on SRO/STO and with the Pt top electrodes.

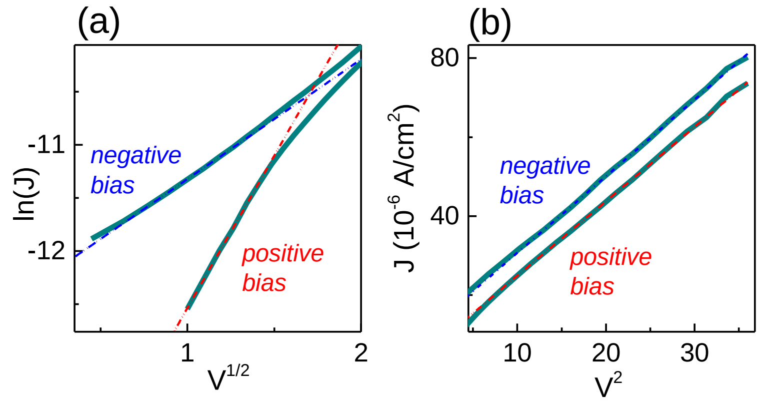

Figure S10. Analysis of density of leakage current J as a function of voltage V using models of (a) Schottky-type carrier injection $\ln J \propto V^{1/2}$ and (b) space charge limited conduction $J \propto V^2$. Straight lines show fits to the models.

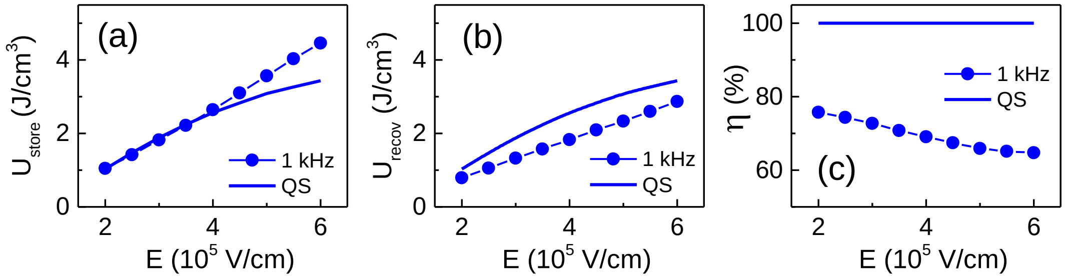

Figure S11. (a) Stored energy density, (b) recoverable energy density, and (c) storage efficiency determined at frequency 1 kHz (solid circles) and in quasi-static conditions (sold curves) in the Pt/BTO SRO/STO capacitor.

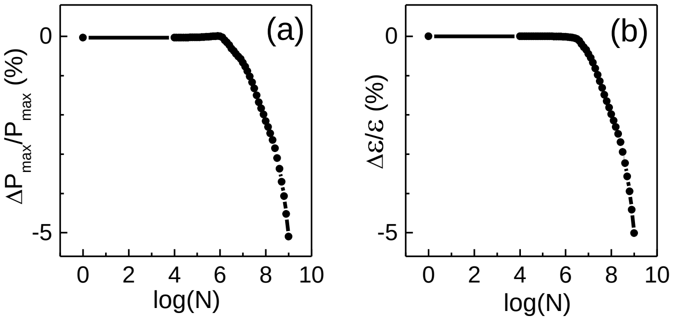

Figure S12. Relative change of (a) maximum polarization and (b) dielectric permittivity as a function of number of switching cycles N.

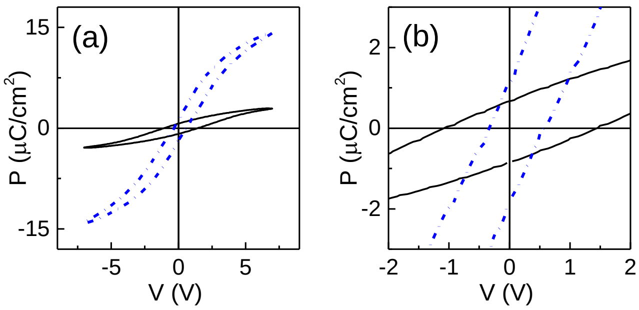

Figure S13. Dynamic polarization-voltage loops measured at room temperature and 1 kHz in the 200-nm-thick polycrystalline BTO film on SRO/Al_2O_3 (solid curves) and 200-nm-thick thermally strained BTO film on SRO/STO (dashed curves).

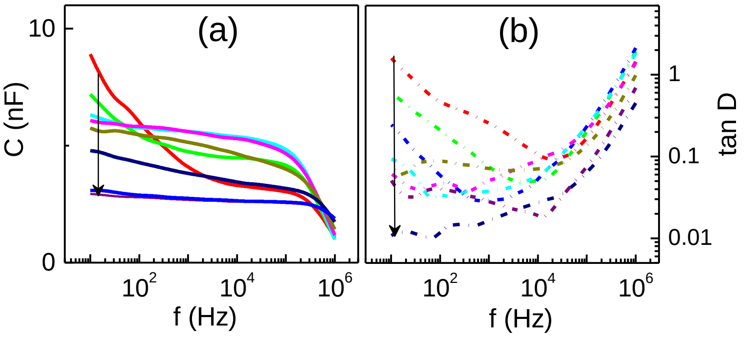

Figure S14. Frequency dispersion of the capacitance and loss factor tan D at the temperatures 450, 400, 350, 300, 250, 200, 150, and 100 K in the SRO/BTO(200nm)/Pt capacitor. Arrows show directions of the temperature decrease.
